# Visual Outcomes and Patient Satisfaction After Implantation of Enhanced Versus Standard Monofocal Intraocular Lenses With a Myopic Target Refraction: A Retrospective Comparative Study

**DOI:** 10.1155/joph/5528302

**Published:** 2026-04-18

**Authors:** Kyung-Sun Na, Jiyoung Emily Lee, Phil Kyu Lee, Hyun Seung Kim, Eun Chul Kim

**Affiliations:** ^1^ Department of Ophthalmology, Yeouido St. Mary’s Hospital, College of Medicine, The Catholic University of Korea, Seoul, Republic of Korea, catholic.ac.kr; ^2^ Department of Ophthalmology, Bucheon St. Mary’s Hospital, College of Medicine, The Catholic University of Korea, Seoul, Republic of Korea, catholic.ac.kr; ^3^ Department of Ophthalmology, Seoul St. Mary’s Hospital, College of Medicine, The Catholic University of Korea, Seoul, Republic of Korea, catholic.ac.kr

**Keywords:** corrected intermediate vision, enhanced monofocal IOL, monofocal IOL, myopic target, uncorrected very near vision

## Abstract

**Purpose:**

To compare the clinical outcomes of enhanced monofocal IOL (Eyhance) and monofocal IOL (TECNIS) implantation in cataract patients whose target refraction is myopia.

**Methods:**

This is a retrospective chart review for patients who underwent cataract surgery and whose target refraction is myopia (−3 D). A total of 68 eyes were enrolled (34 patients enrolled: enhanced monofocal (Group 1) = 32 eyes, monofocal (Group 2) = 36 eyes; bilateral IOL implantation in each patient). For the primary endpoint, uncorrected and corrected near (UNVA and CNVA), intermediate (UIVA and CIVA), and distant (UDVA and CDVA) visual acuities; manifest refraction spherical equivalent (MRSE); and satisfaction score were evaluated before and 3 months after surgery.

**Results:**

Postoperative CIVA was significantly better in Group 1 (0.09 ± 0.05 logMAR) compared to Group 2 (0.21 ± 0.22 logMAR; mean difference: 0.12 logMAR; *p* < 0.05). UNVA at 20 cm was also better in Group 1 (0.12 ± 0.14 logMAR) than in Group 2 (0.22 ± 0.14 logMAR; *p* < 0.05). Defocus testing identified between‐group differences at −4.0 D without distant correction and at −1.5 to −2.0 D with distant correction, favoring Group 1 (*p* < 0.05). Overall patient satisfaction was significantly higher in Group 1 (*p* < 0.05), whereas contrast sensitivity did not differ significantly between the groups.

**Conclusion:**

Enhanced monofocal IOL with a myopic target showed better corrected intermediate vision and uncorrected very near vision at 20 cm compared with monofocal IOL. Overall satisfaction with enhanced monofocal IOL is also higher than with monofocal IOL.

## 1. Introduction

One of the most popular and successful surgical treatments in the world is cataract extraction and implantation of intraocular lenses (IOLs) [1], and the cataract surgical rate has been steadily increasing, particularly in Korea [2]. Multiple advances in IOLs are being employed to improve not only postoperative distant vision but also near vision in cataract patients [3].

Patients who have long depended on uncorrected near vision often do not prefer full emmetropia after cataract surgery, because it changes their habitual working distance and increases the need for spectacles during near tasks [4]. Myopic patients with −3.0 diopters (D) of refractive power often manage daily household tasks without spectacles. Their preference for removing glasses during reading or other routine daily activities reflects a desire to maintain their habitual visual comfort at near distances. [5]. Consequently, some patients with preoperative myopia prefer to remain myopic after surgery to continue reading without spectacles [6]. This preference often corresponds to a desire to maintain uncorrected near vision at a habitual reading distance, typically around 33 cm, which equates to a refractive target of −3.0 D. Indeed, recent expert consensus guidelines recommend that patients with habitual uncorrected near vision due to myopia be counseled regarding the option of intentional residual myopia after surgery [4]. However, cataract surgery with a myopic target significantly increases spectacle dependency in distant vision [7]. An alterative approach is mini‐monovision, where emmetropia is targeted in the dominant eye and a small degree of myopia is intentionally left in the nondominant eye, usually around −0.75 to −1.75 D. This procedure is usually met with satisfaction by patients who report good outcomes in near and distant vision [7, 8]; however, it reduced stereopsis, and not every patient tolerates interocular refractive differences equally [9].

With patients increasingly relying on vision at more than one working distance, cataract surgery has moved beyond simply restoring far vision, prompting wider use of IOLs with enhanced functional range [10]. Multifocal IOL has shown excellent outcomes in improving near and intermediate vision without losing far vision, but it can cause the unwanted side effect of halos and glare [11–14]. To enhance functional intermediate acuity, an enhanced monofocal IOL featuring a continuous power distribution and a re‐engineered aspheric anterior surface was recently introduced [15–17]. This IOL is engineered to provide the same high level of visual quality as a standard monofocal IOL, ensuring that patients do not experience increased complications such as glare and halos while gaining better intermediate functionality [18]. Although previous studies have suggested that enhanced monofocal IOLs provide better intermediate visual outcomes than standard monofocal IOLs, it might not be enough to classify enhanced monofocal IOLs as extended depth of focus (EDoF) IOLs because of the insufficient range of vision [19]. It yields greater difficulty with near vision compared to multifocal or EDoF IOL [20].

Several studies have reported visual outcomes from enhanced monofocal IOL with an emmetropic target [15, 21, 22]. However, as far as we have ascertained, no studies have compared the visual efficacy of enhanced monofocal IOL and monofocal IOL with a myopic target.

Thus, we compared the outcomes of enhanced monofocal IOL and monofocal IOL implantation in cataract patients whose target refraction is myopia.

## 2. Methods

We retrospectively reviewed the medical records of patients who received cataract surgery at Bucheon St. Mary’s Hospital between January 2021 to December 2022. Adhering to the tenets of the Declaration of Helsinki, this research involved a retrospective analysis of clinical records. The protocol received formal approval from the Institutional Review Board (IRB)/Ethics Committee of Bucheon St. Mary’s Hospital (HC23RISE0095).

### 2.1. Selection Criteria for Study Participants

We identified and screened 68 eyes (34 participants) diagnosed with cataracts for this retrospective analysis. The inclusion criteria were patients who had myopia of more than −3.0D before being diagnosed with cataracts and a desired postoperative target refraction of −3.0D, based on discussion with the surgeon regarding lifestyle and near vision preferences.

Exclusion criteria comprised ocular comorbidities that might compromise postoperative visual quality, including high corneal astigmatism (> 1.0 D), keratoconus, or other ectatic and degenerative corneal disorders. Furthermore, eyes with zonular instability (e.g., lens subluxation or dislocation), amblyopia, or significant vitreoretinal pathologies were also excluded from the study. There was no upper limit for preoperative myopia as long as other ocular pathologies were absent. The general framework for patient selection and data collection was adapted from our previous methodology [23, 24].

### 2.2. Patient Examination

Patients received sequential bilateral cataract surgery with a TECNIS‐enhanced monofocal IOL (ICB, Group 1: 32 eyes, Johnson & Johnson Vision, Irvine, CA, USA) or a TECNIS monofocal IOL (ZCB, Group 2: 36 eyes). This was a retrospective study, and IOL allocation was not randomized. Patients were allocated to Group 1 or Group 2 by IOL types, which were determined based on IOL availability and patient preference during the consultation period, representing a consecutive series. A comprehensive ophthalmic evaluation was performed for every participant to establish a precise clinical baseline. We documented all demographic characteristics and perioperative clinical parameters.

The following examinations were performed preoperatively and postoperatively after one day, one week, and one, two, and three months: Uncorrected and corrected distant visual acuities (UDVA and CDVA), with results converted to logMAR scales for standardized analysis, manifest refraction, ocular biometry, and keratometry, were assessed using the IOLMaster 700 partial coherence interferometry device (Carl Zeiss Meditec AG), and corneal topographic analysis was performed with Pentacam (Oculus, Germany). A thorough anterior segment examination using a slit lamp was also conducted for all participants.

Preoperatively, fundus examination with dilated pupils was performed. The IOL power was determined based on a predefined refractive target of −3.0 D, utilizing the SRK/T formula to accommodate the specific visual requirements of myopic patients, as this was the standard institutional protocol at the time of the surgeries.

Postoperatively at 3 months, uncorrected and corrected intermediate visual acuities (UIVA and CIVA) and near visual acuities (UNVA and CNVA) were measured; intermediate visual acuities were measured at 66 cm, while near visual acuities were measured at 33 cm and 20 cm. Monocular defocus curves were measured under photopic conditions (85 cd/m^2^) and performed in 0.50 D steps from +1.0 D to −4.0 D. Patient‐reported outcomes were gathered three months postoperatively using the validated Cataract TyPE Specification questionnaire to quantify subjective satisfaction levels [24]. This questionnaire, previously validated in Korea, assesses satisfaction using a 5‐point Likert scale (where 1 = very satisfied, 5 = very unsatisfied) across several domains, including overall satisfaction, far vision, near vision, glare, and halos. Contrast sensitivity was measured using the CSV‐1000 chart (VectorVision, USA) under photopic (85) and mesopic (3 cd/m^2^) conditions.

### 2.3. Surgical Procedures

Phacoemulsification procedures were executed by one experienced surgeon (E.C.K.) utilizing the CENTURION Vision System (Alcon Laboratories, Inc., Fort Worth, TX, USA). Topical anesthesia was administered to all subjects prior to the initial clear corneal incision of 2.75 mm, which was positioned along the steep corneal meridian. Following a 6.0 mm circular capsulotomy marked with gentian violet, standard hydromaneuvers including dissection and delineation were conducted. Following phacoemulsification, a clear IOL was carefully positioned within the capsular bag. The corneal incisions were closed via stromal hydration without the need for sutures. The interval between each eye operation for each patient was one week. Complicated surgeries such as small pupil or posterior capsule rupture were discarded from the analysis.

After surgery, Gatiflo (0.3% gatifloxacin; Taejun, Korea) and Predbell (1% prednisolone acetate; Chong Kun Dang, Korea) were administered four times daily for one month and then discontinued. Ocular examination was performed postoperatively after one day, one week, and one, two, and three months.

### 2.4. Statistical Analyses

Data were analyzed with SPSS software for Windows (Version 21.0.1; SPSS Inc., Chicago, IL, USA). To evaluate changes in VA and refraction within each group, we applied the Wilcoxon signed‐rank test for longitudinal comparisons between baseline and the 3‐month postoperative follow‐up. Comparisons between the two groups were performed with the Mann–Whitney test. To assess the magnitude of the differences between groups, effect sizes (Cohen’s d) and 95% confidence intervals (CIs) for the mean differences (MDs) were calculated. *p* values < 0.05 were considered statistically significant.

## 3. Results

Patient preoperative data for each group are shown in Table 1. No statistically significant between‐group difference was observed according to age, preoperative manifest refraction spherical equivalent (MRSE), UDVA, or CDVA (*p* > 0.05).

**TABLE 1:** Preoperative data of patients.

Parameter	Group 1 (enhanced monofocal)	Group 2 (monofocal)	*p* value
Mean age (years)	57.7 ± 9.7	62.0 ± 11.5	0.155
Keratometer (D)	43.25 ± 2.12	43.53 ± 2.37	0.431
Refractive astigmatism (D)	0.53 ± 0.21	0.51 ± 0.19	0.209
MRSE (D)	−6.09 ± 3.59	−6.50 ± 3.97	0.847
Axial length (mm)	25.73 ± 1.73	25.59 ± 1.68	0.422
UDVA (logMAR)	0.58 ± 0.17	0.52 ± 0.32	0.862
CDVA (logMAR)	0.44 ± 0.28	0.45 ± 0.26	0.798
Eyes (n)	32	36	

*Note:* No statistical difference was observed between the two groups (*p* > 0.05). Values are presented as mean ± SD.

Abbreviations: CDVA, corrected distant visual acuity; D, diopter; logMAR, logarithm of the minimum angle of resolution; MRSE, manifest refraction spherical equivalent; UDVA, uncorrected distant visual acuity.

### 3.1. Assessment of Visual Outcomes After Surgery

Clinical equivalence was observed between the two study groups regarding most postoperative metrics. Specifically, no statistically significant disparities were identified in terms of postoperative UDVA, CDVA, UIVA, UNVA at 33 cm, CNVA at 20 cm, CNVA at 33 cm, or MRSE 3 months after cataract surgery (*p* > 0.05). However, CIVA and UNVA at 20 cm in Group 1 (0.09 ± 0.0 and 0.12 ± 0.14 logMAR, respectively) were significantly better than Group 2 (0.21 ± 0.22 and 0.22 ± 0.14, respectively; CIVA: MD −0.12 logMAR, 95% CI: −0.20 to −0.04, Cohen’s *d* = 0.75; UNVA at 20 cm: MD ‐0.10 logMAR, 95% CI: −0.17 to −0.03, Cohen’s *d* = 0.71; all *p* < 0.05; Table 2).

**TABLE 2:** Postoperative visual outcomes at 3 months after surgery.

Parameter	Group 1 (enhanced monofocal)	Group 2 (monofocal)	*p* value
UDVA (logMAR)	0.63 ± 0.33	0.59 ± 0.45	0.862
CDVA (logMAR)	0.03 ± 0.02	0.02 ± 0.02	0.719
UIVA (logMAR)	0.44 ± 0.25	0.49 ± 0.37	0.409
CIVA (logMAR)	0.09 ± 0.05	0.21 ± 0.22	0.030^∗^
UNVA at 20 cm (logMAR)	0.12 ± 0.14	0.22 ± 0.14	0.014^∗^
UNVA at 33 cm (logMAR)	0.34 ± 0.21	0.37 ± 0.20	0.298
CNVA at 20 cm (logMAR)	0.72 ± 0.44	0.74 ± 0.53	0.582
CNVA at 33 cm (logMAR)	0.57 ± 0.32	0.61 ± 0.35	0.605
MRSE (D)	−2.79 ± 0.58	−3.04 ± 1.31	0.695
Eyes (*n*)	32	36	

*Note:* The postoperative CIVA and UNVA at 20 cm of Group 1 exhibited a significant advantage over those of Group 2 (*p* < 0.05). UNVA at 20, 33 cm, uncorrected near visual acuity at 20, 33 cm; CNVA at 20, 33 cm, corrected near visual acuity at 20, 33 cm. Values are presented as mean ± SD.

Abbreviations: CDVA, corrected distant visual acuity; CIVA, corrected intermediate visual acuity; D, diopter; logMAR, logarithm of the minimum angle of resolution; UDVA, uncorrected distant visual acuity; UIVA, uncorrected intermediate visual acuity.

^∗^ *p* < 0.05. Significant differences were observed in CIVA (mean difference [MD]: −0.12 logMAR, 95% CI: −0.20 to −0.04, Cohen’s *d* = 0.75) and UNVA at 20 cm (MD: −0.10 logMAR, 95% CI: −0.17 to −0.03, Cohen’s *d* = 0.71).

### 3.2. Defocus Curve

**FIGURE 1:**
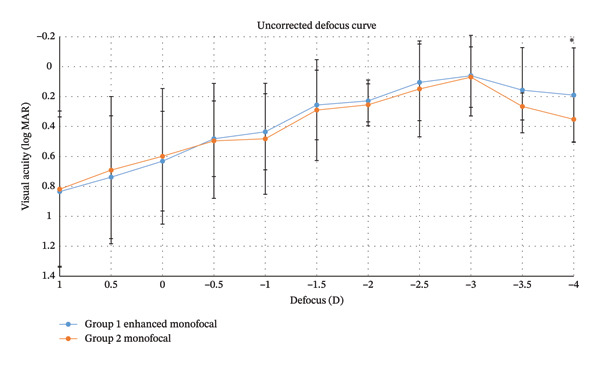
Mean defocus curves of the two intraocular lens groups without distant correction. Both curves showed a peak at defocus −3.0 D and a reduction in visual acuity with the increase in negative defocus and positive defocus. Group 1 provided significantly better defocus results at −4.0 D of defocus range compared to Group 2 (*p* < 0.05). logMAR = logarithm of the minimum angle of resolution. ^∗^ *p* < 0.05. Values are presented as mean ± SD.

Figure 1 shows the uncorrected defocus curves for both groups at 3 months postoperatively. In both groups, the best visual acuity was observed at −3.0 D, with a gradual decrease as the defocus level moved away from this point in either direction. However, Group 1 showed significantly better defocus results at −4.0 D in the defocus range compared to Group 2 (*p* < 0.05; Figure 1).

**FIGURE 2:**
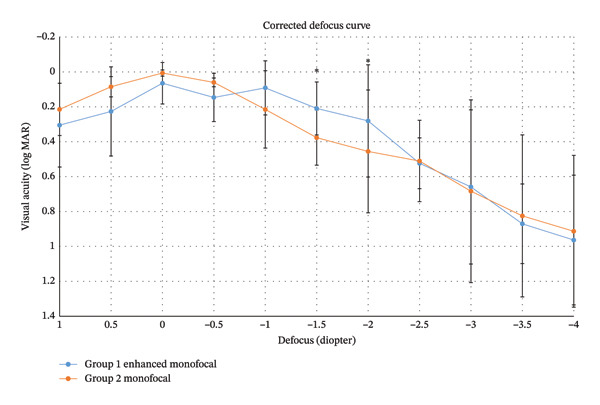
Mean defocus curves of the two intraocular lens groups with distant correction. Both curves showed a peak at defocus 0.00 D (4 m) and a reduction in visual acuity with the increase in negative defocus. However, Group 1 provided significantly better defocus results at −1.5 D and −2.0 D of defocus range (corresponding to 66 ∼ 50 cm) compared to Group 2 (*p* < 0.05). logMAR = logarithm of the minimum angle of resolution. ^∗^ *p* < 0.05. Values are presented as mean ± SD.

Figure 2 shows the defocus curves measured with distant correction in both groups three months after cataract surgery. Both groups achieved their best visual acuity at 0.00 D (4 m), with acuity gradually declining toward increasing negative defocus level. However, Group 1 exhibited significantly enhanced visual outcomes within the intermediate range. While Group 2 showed a sharper drop‐off in visual acuity, Group 1 maintained higher resolution at defocus levels of −1.5 D and −2.0 D (equivalent to 66–50 cm), providing a clear clinical advantage for intermediate‐distance tasks (*p* < 0.05; Figure 2).

### 3.3. Contrast Sensitivity

No statistically significant differences were observed between the groups according to photopic and mesopic contrast sensitivity at any spatial frequency at postoperative three months (*p* > 0.05; Figure 3).

FIGURE 3Photopic and mesopic contrast sensitivity at postoperative 3 months. There were no significant differences between the two groups according to photopic and mesopic contrast sensitivity at postoperative 3 months (*p* > 0.05). Values are presented as mean ± SD.

**Figure:**
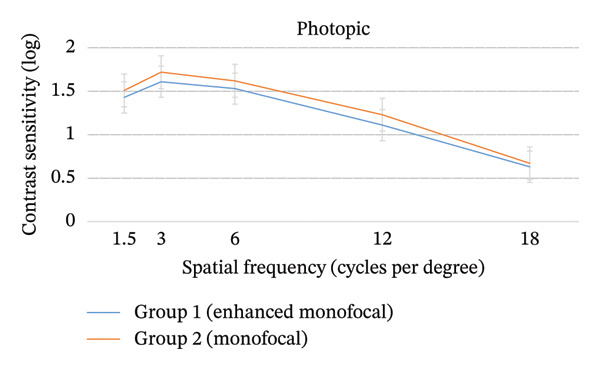
(a)

**Figure:**
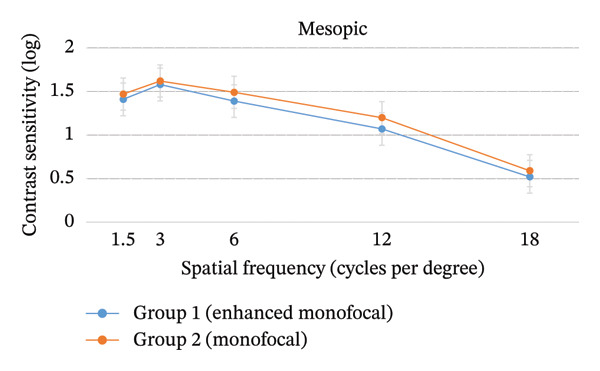
(b)

### 3.4. Patient‐Reported Outcomes and Satisfaction Analysis

Statistical analysis of patient‐reported outcomes revealed no discernible disparities between the two cohorts regarding satisfaction of far and near vision or photic phenomena, such as glare and halo, between the two groups (*p* > 0.05). However, Group 1 had a significantly better overall satisfaction score than Group 2 (1.50 ± 0.21 for Group 1, 1.77 ± 0.35 for Group 2; *p* < 0.05; Table 3).

**TABLE 3:** Subjective satisfaction score.

Parameter	Group 1 (enhanced monofocal)	Group 2 (monofocal)	*p* value
Overall satisfaction	1.50 ± 0.21	1.77 ± 0.35	0.001^∗^
Far vision satisfaction	1.48 ± 0.44	1.58 ± 0.45	0.084
Near vision satisfaction	1.25 ± 0.23	1.29 ± 0.19	0.776
Glare and halo	1.15 ± 0.24	1.30 ± 0.29	0.067

*Note:* High score means low satisfaction. Overall satisfaction of Group 1 exhibited a significant advantage over that of Group 2 (*p* < 0.05). Values are presented as mean ± SD.

^∗^ *p* < 0.05.

## 4. Discussion

Multifocal IOL not only delivers comparable distance visual acuity and better near visual acuity than monofocal IOL but also yields lower spectacle dependence [25]. However, multifocal IOL also resulted in more problems with glare and halos compared to monofocal IOL [25]. To improve near vision with monofocal IOL, bilateral monofocal IOLs implanted with a mild myopic target offered better intermediate vision than bilateral emmetropia [10].

The TECNIS Monofocal ZCB00 IOL is made from an ultraviolet light−blocking hydrophobic material not associated with glistenings that have been widely implanted throughout the world and which yield well‐known outcomes [26]. The TECNIS Eyhance ICB00 IOL (Johnson & Johnson) is an enhanced monofocal refractive lens with a modified aspheric anterior surface with increasing power distribution from the periphery to the center that differs from that of its monofocal ZCB00 predecessor (Johnson & Johnson) [27]. It provides better intermediate vision than monofocal IOL, such as when working on a computer, smartphone, or car dashboard, and also offers similar distance performance [28]. However, because near vision is not sufficient for near tasks with enhanced monofocal IOL, the mini‐monovision technique was developed to improve near vision [29]. The mini‐monovision group showed better binocular uncorrected near vision and higher spectacle independence rates compared to the emmetropia group [29].

For highly and moderately myopic patients, target refraction is the most critical issue, and the target should be acceptable to each patient’s preoperative lifestyle [30]. Hayashi and Hayashi reported that a monofocal IOL simulating −2.0 D of myopia provided adequate visual acuity for both near and intermediate vision [30]. However, some myopic patients just want to wear glasses after cataract surgery, like they used to before their operation.

This study evaluated the depth of focus and the range of functional vision in patients implanted with an enhanced monofocal IOL targeting myopia. Our results demonstrated that the enhanced monofocal IOL provided significantly better postoperative CIVA (0.09 ± 0.05 logMAR) compared to the standard monofocal IOL (0.21 ± 0.22; *p* < 0.05). In the defocus curve analysis, the enhanced monofocal group showed superior visual outcomes at −4.0 D without distant correction and at −1.5 D to −2.0 D with distant correction (corresponding to 66 cm−50 cm) compared to standard monofocal IOLs (*p* < 0.05; Figure 2). Notably, the −4.0 D point in the uncorrected defocus curve corresponds to the intermediate vision range when corrected for a myopic target of −3.0 D. These findings suggest that the enhanced monofocal IOL effectively extends the range of vision to the intermediate distance without compromising the near vision benefits of the myopic target. This expands upon previous findings that the enhanced monofocal IOL provided higher monocular and binocular UIVA in the emmetropic target compared to monofocal IOL [18].

Patients who have an implanted monofocal IOL with a myopic target should remove their glasses when performing near and even intermediate work, but patients who have an implanted enhanced monofocal IOL with a myopic target do not need to remove their glasses when doing distant and intermediate work, such as dashboard viewing.

We also evaluated the range of uncorrected near vision in the enhanced monofocal IOL group. There were no significant differences between the two groups according to postoperative UIVA or UNVA at 33 cm (*p* > 0.05), but UNVA at 20 cm in Group 1 (0.12 ± 0.14 logMAR) was significantly better than in Group 2 (0.22 ± 0.14; *p* < 0.05; Table 2). We suggest that enhanced monofocal IOL has a wider range, even in near vision, than monofocal IOL because of the wide depth of focus. Therefore, enhanced monofocal IOL with a myopic target may provide better uncorrected near vision at 20 cm (such as when looking at a hand mirror or smartphone) than monofocal IOL.

In this study, overall patient satisfaction was significantly higher in Group 1 (1.50 ± 0.21) compared to Group 2 (1.77 ± 0.35; *p* < 0.05; Table 3). This higher satisfaction is likely attributed to the extended depth of focus provided by the enhanced monofocal IOL, which offers a continuous range of functional vision from near to intermediate distances [28]. Unlike standard monofocal IOLs, where vision may drop‐off sharply beyond the focal point, the enhanced monofocal IOL appears to provide better visual quality for various daily activities. Notably, a recent study reported that the enhanced monofocal IOL demonstrated overall satisfaction comparable to that of trifocal or extended depth of focus IOLs, despite being a monofocal platform [31]. Furthermore, there were no significant differences between the groups according to photopic and mesopic contrast sensitivity at any spatial frequency at postoperative three months (*p* > 0.05; Figure 3). These findings confirmed that the modified aspheric anterior surface of the enhanced monofocal IOL maintains optical quality comparable to that of a standard monofocal IOL, avoiding the reduction in contrast sensitivity often associated with diffractive multifocal IOLs [15, 27, 28].

If target refraction were −1.75 to −2.0D (intermediate distance) instead of −3.0D (near distance) in monofocal IOL and enhanced monofocal IOL, the quality‐of‐life outcomes from this study would have been different. Patients who have an implanted monofocal IOL with an intermediate myopic target should remove their glasses when doing intermediate work only. They may need two kinds of glasses for distant and near vision, but patients who have implanted an enhanced monofocal IOL with an intermediate myopic target do not need another pair of glasses when doing near and intermediate work because of its wide range of vision. They may need just one pair of glasses for distant vision. It is not necessary to implant an enhanced monofocal IOL with an intermediate myopic target because it already provides good intermediate vision with an emmetropic target.

As far as we have ascertained, this is the first study to compare the clinical outcomes of enhanced monofocal IOL and monofocal IOL implantation in cataract patients whose target refraction is myopia.

Our findings are subject to several limitations, notably the single‐center nature of the study and the limited number of enrolled eyes. Subsequent multicenter investigations with expanded patient populations are necessary to validate the long‐term visual performance and patient satisfaction of enhanced monofocal IOL and monofocal IOL implantation in cataract patients whose target refraction is myopia.

In conclusion, enhanced monofocal IOL with a myopic target was associated with better corrected intermediate vision and uncorrected very near vision at 20 cm compared to monofocal IOL. Overall satisfaction with enhanced monofocal IOL was also higher than with monofocal IOL. These findings suggest that enhanced monofocal IOLs may provide a functional advantage over standard monofocal IOLs for cataract patients desiring a myopic target.

## Author Contributions

Kyung‐Sun Na was involved in the analysis and interpretation of data and drafting the manuscript. Kyung‐Sun Na, Jiyoung Emily Lee, Phil Kyu Lee, Hyun Seung Kim, and Eun Chul Kim made contributions to the acquisition of data and drafting. Eun Chul Kim contributed to conception and design, analysis and interpretation of data, and drafting and revising the manuscript.

## Funding

This study was supported by grants from the Basic Science Research Program through the National Research Foundation of Korea, funded by the Ministry of Education (2022R1A2C2006109).

## Disclosure

All authors read and approved the final manuscript.

## Ethics Statement

This study was approved by the Institutional Review Board (IRB) of Bucheon St. Mary Hospital (No. HC23RISE0095); the informed consent was waived. All clinical investigations have been conducted according to the principles expressed in the Declaration of Helsinki.

## Consent

Please see the Ethics Statement.

## Conflicts of Interest

The authors declare no conflicts of interest.

## Data Availability

The datasets used and/or analyzed during the current study are available from the corresponding author on reasonable request.
